# Health inequalities in PM₂.₅-related symptoms during Thailand’s haze season: the role of education, sociodemographic, lifestyle, protective behaviors, and environmental factors

**DOI:** 10.3389/fpubh.2026.1899785

**Published:** 2026-10-01

**Authors:** Nalin Sittitoon, Wuttipat Kiratipaisarl, Ratana Sapbamrer

**Affiliations:** 1 School of Environmental Health, Institute of Public Health, Suranaree University of Technology, Nakhon Ratchasima, Thailand; 2 Department of Community Medicine, Faculty of Medicine, Chiang Mai University, Chiang Mai, Thailand; 3 Environmental and Occupational Medicine Excellence Center (EnOMEC), Faculty of Medicine, Chiang Mai University, Chiang Mai, Thailand

**Keywords:** acute health symptom, air pollution, education, health inequalities, protective behavior, sociodemographic

## Abstract

**Background:**

Seasonal haze pollution in Thailand exposes populations to recurrent fine particulate matter (PM₂.₅), yet its health impacts are unequally distributed, reflecting disparities in education, social vulnerability, and protective capacity. This study examined the associations of educational attainment, sociodemographic, lifestyle, protective behaviors, and environmental factors with acute health symptoms during the haze season.

**Methods:**

A nationwide cross-sectional study was conducted between January and April 2025 among 3,916 adults across Thailand. Data were collected through standardized interviews assessing sociodemographic characteristics, protective behaviors, and acute health symptoms. Provincial PM₂.₅ concentrations were obtained from satellite-based monitoring data. Latent class analysis was used to identify protective behavioral phenotypes, and mixed-effects multivariable logistic regression models were applied to evaluate determinants of health symptoms.

**Results:**

Mean monthly PM₂.₅ concentrations exceeded international guideline values throughout the study period. Respiratory symptoms were the most prevalent (80.7%), followed by eye (52.8%), dermatological (48.7%), and neurological symptoms (33.1%). Three behavioral phenotypes were identified: compliers (33.4%), non-compliers (48.6%), and a neutral group (18.0%). Higher PM₂.₅ exposure was independently associated with increased odds of respiratory and dermatological symptoms. Compared with compliers, non-compliers and neutral participants had significantly higher odds of respiratory and neurological symptoms. Female sex, younger age, comorbidity, alcohol consumption, urban or suburban residence, and lack of village health volunteer engagement were also associated with elevated symptom risk.

**Conclusion:**

Higher ambient PM₂.₅ exposure, non-compliant protective behavioral phenotypes were associated with increased odds of acute health symptoms, highlighting persistent social inequalities in vulnerability to haze pollution. Equity-oriented public health strategies that strengthen community health resources, promote protective behaviors, and address educational disparities may help reduce unequal vulnerability to air pollution.

## 1 Introduction

Seasonal haze pollution poses a persistent and escalating public health challenge in Thailand, particularly during the dry season from January to April, when fine particulate matter (PM₂.₅) concentrations frequently exceed national standards and World Health Organization guideline values (1–3). These recurrent pollution episodes arise from a complex interplay of biomass burning, agricultural residue combustion, forest fires, transboundary transport, and unfavorable meteorological conditions that constrain pollutant dispersion (1, 3, 4). Consequently, large segments of the Thai population experience repeated exposure to elevated PM₂.₅ concentrations each year, contributing to a substantial cumulative burden of morbidity and mortality nationwide (2, 5, 6). A growing body of evidence identifies PM₂.₅ as a major environmental risk factor for adverse health outcomes. Fine particles can penetrate deeply into the respiratory tract and enter systemic circulation, triggering oxidative stress, inflammation, immune dysregulation, mitochondrial dysfunction, and epigenetic alterations (7–9). Although respiratory outcomes have been most extensively studied, emerging evidence indicates that acute PM₂.₅ exposure also affects the eyes, skin, and nervous system (8, 10–13). These multisystem effects reflect both direct contact of pollutants and indirect systemic pathways mediated by multiple biological mechanisms (14). However, population-based evidence capturing this broader spectrum of acute health symptoms during haze episodes remains limited, particularly in low- and middle-income settings.

Importantly, the health impacts of haze exposure are not uniform across populations. Individual vulnerability is shaped not only by ambient pollution concentrations but also by sociodemographic characteristics, lifestyle behaviors, and the protective responses adopted during haze episodes (15–18). In Thailand, public health advisories commonly recommend protective behaviors such as mask use, limiting outdoor activities, improving indoor air quality, and avoiding pollution-generating activities (19). However, adherence to these recommendations varies widely. Previous studies suggest that individuals tend to preferentially adopt low-cost, easily implementable behaviors, whereas practices requiring financial investment, infrastructure, or substantial lifestyle modification are less frequently adopted (20–22). Such heterogeneity in behavioral responses may substantially modify the effective dose of inhaled pollutants and, consequently, acute health risk.

Despite increasing recognition of behavioral determinants, most haze-related studies assess protective behaviors in isolation, focusing on single practices or composite scores (23). These approaches may overlook how protective behaviors cluster in real-world settings, where individuals simultaneously adopt or neglect multiple practices. Latent class analysis provides a person-centered approach to identifying distinct behavioral phenotypes that more accurately reflect real-world behavior patterns. Understanding these phenotypes is particularly important in the context of haze exposure; however, empirical evidence linking behavioral phenotypes to multisystem acute health symptoms under PM₂.₅ exposure remains limited, especially at the national level in Thailand. Moreover, environmental context may further shape both exposure and health responses. Urban, suburban, and rural areas differ markedly in dominant emission sources, activity patterns, and exposure profiles, potentially contributing to distinct symptom patterns during haze episodes (1, 24). Nevertheless, few nationwide studies have simultaneously examined environmental context, behavioral responses, and health outcomes.

To address these gaps, we conducted a nationwide cross-sectional study in Thailand during the 2025 haze season to examine the associations between ambient PM₂.₅ exposure, protective behavioral phenotypes, and acute multisystem health symptoms. Specifically, we aimed to (1) characterize patterns of haze-related protective behaviors using latent class analysis, (2) assess the prevalence of acute respiratory, eye, dermatological, and neurological symptoms, and (3) evaluate the associations of educational attainment, sociodemographic, lifestyle, protective behaviors, and environmental factors with acute health symptoms during the haze season. This study provides comprehensive evidence to support behaviorally informed, context-specific, and equity-oriented strategies for mitigating the health impacts of recurrent haze pollution in Thailand.

## 2 Methods

### 2.1 Setting and study population

This nationwide cross-sectional study was conducted between January and April 2025 to assess behavioral phenotypes and acute health symptoms during the haze season in Thailand. The target population included Thais aged 20–80 years living in the Central, Southern, Northern, and North-Eastern regions, which are defined by geographical, climatic, cultural, and administrative characteristics. Eligible participants were individuals who had lived in their respective provinces for at least 6 months prior to the survey. Individuals unable to provide informed consent or complete interviews were excluded. The total population in this age range across the four regions was 50,259,780, 17,615,707 in the Central, 6,833,827 in the South, 9,165,338 in the North, and 16,644,908 in the North-East (25). Using EpiInfo with an expected frequency of 13.9%, a 99.9% confidence level, a 2% margin of error, and four clusters, the minimum required sample size was 3,240 (26). Population-proportional sampling was applied to ensure regional representativeness and reduce selection bias. Data were collected from 72 of 77 provinces, including 27 in the Central, 12 in the South, 15 in the North, and 18 in the North-East. In total, 4,271 participants were initially recruited and consented; listwise complete data related to all determinants was found in 3916 participants (91.7%). Due to low proportion of missing data, the missing-completely-at-random assumption was applied for all analysis. The final sample comprised 1,421 participants from the Central region, 492 from the South, 679 from the North, and 1,324 from the North-East. A choropleth map of participant distribution across Thailand is presented in Figure 1. The distribution of participants by province is presented in Supplementary Table S1.

**Figure 1:**
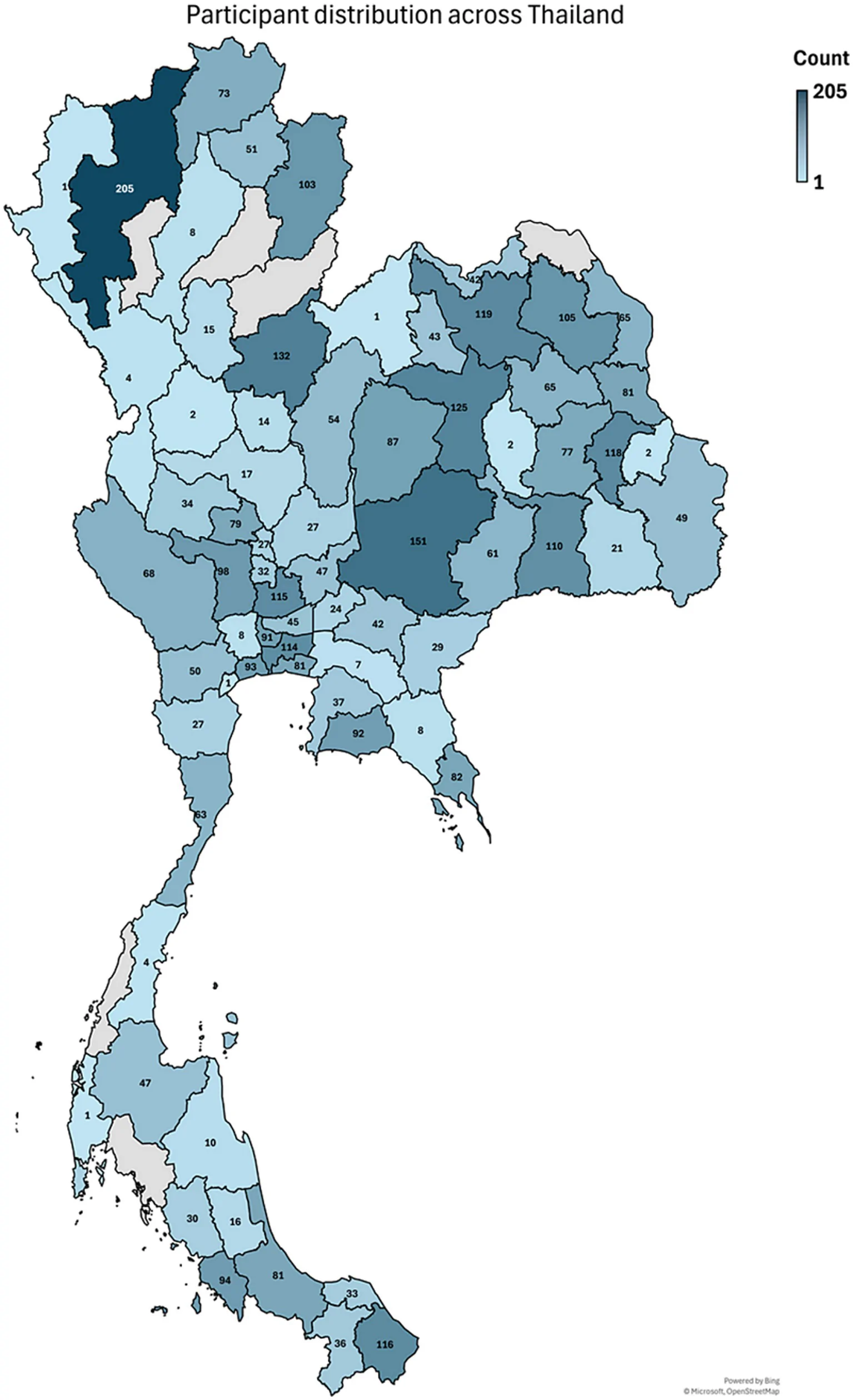
A choropleth map of participant distribution across Thailand.

### 2.2 Interviews

A structured face-to-face interview served as the primary data collection method. To accommodate Thailand’s linguistic and cultural diversity, 72 interviewers, one from each participating province, were recruited from local healthcare personnel and health science students. All were native to their provinces and fluent in regional languages, ensuring effective communication and minimizing cultural or linguistic bias. Prior to data collection, all interviewers completed standardized training provided by the research team. Each interview took approximately 10–15 min. Standardized face-to-face interviews and trained interviewers were used to reduce misclassification. Selection bias was minimized through population-proportional sampling across regions. Residual confounding may persist despite multivariable adjustment.

The questions on the interview form were systematically structured into three key sections, including sociodemographic characteristics, health behavior, and acute health symptoms during the haze season. Sociodemographic data included age (years), gender (male or female), region (Central, South, North, or North-East), education level (no formal education, primary education, secondary school, Bachelor’s degree, or postgraduate degree), monthly income (≤450 USD, 451–1,000 USD, 1,001–1,500 USD, or > 1,500 USD), status of living area (rural, suburban, or urban), cigarette smoker (yes or no), alcohol consumption (yes or no), co-morbidity such as hypertension, cardiovascular disease, diabetes, chronic lung disease, kidney disease, and obesity (yes or no), village health volunteer participation (yes or no), routine health checkup (yes or no), and type of mask use during haze season (cloth or surgical mask or N95 respirator).

The section on health behaviors during the haze season was developed based on established frameworks from guidelines issued by the Thai Ministry of Public Health (2025) (19) and relevant literature (23, 27). An initial pool of items was drafted by three experts in public health, medicine, and environmental health. The questionnaire then underwent two rounds of expert review with five specialists from these fields, who provided recommendations to improve item clarity, relevance, and content validity. The final Thai questionnaire included 19 items, each rated on a three-point Likert scale (never, sometimes, always).

The section on acute health symptoms assessed participants’ respiratory, eye, dermatological, and neurological symptoms experienced during the month preceding the interview, which was conducted between January and April 2025. Thus, symptom reporting corresponded to the same monthly period used for ambient PM₂.₅ exposure assessment. The respiratory symptoms included coughing, nasal congestion, runny nose, dry throat, phlegm, sore throat, dyspnea, shortness of breath, wheezing, asthma, sinusitis, and nosebleed. The eye symptoms include eye itchiness, burning eyes, eye tearing, dry eyes, and conjunctivitis. The skin symptoms include acne, white/red rashes, atopic dermatitis, and white/red pimps. The neurological symptoms included headache and migraine. Participants were considered to have acute health symptoms of those systems if they had at least one symptom from each system.

The validity and reliability of the interview form were evaluated prior to data collection. All items demonstrated strong content validity, with Index of Congruence (IOC) scores exceeding the accepted threshold (>0.5) and ranging from 0.87 to 0.99. In terms of reliability, the instrument showed high internal consistency, with a Cronbach’s alpha coefficient of 0.840. Moreover, the interview form demonstrated excellent measurement stability, with intra-rater and inter-rater reliability coefficients of 0.976 and 0.967, respectively.

### 2.3 Monthly ambient PM₂.₅ levels during the haze season

Between 1 January and 30 April 2025, corresponding to Thailand’s annual haze season, daily ambient PM₂.₅ concentrations for all provinces were obtained from the publicly available monitoring system of the Geo-Informatics and Space Technology Development Agency (GISTDA), Thailand (28). GISTDA generates nationwide satellite-derived PM₂.₅ estimates by integrating Aerosol Optical Depth (AOD) observations with measurements from the national ground-based air quality monitoring network. The monitoring system provides continuous nationwide spatial coverage at a resolution of up to 1 × 1 km, enabling estimation of ambient PM₂.₅ concentrations in areas without ground monitoring stations. According to GISTDA, the satellite-derived estimates are continuously refined through comparison with ground monitoring observations, and the current operational model has an average prediction error of approximately 4–5 μg/m^3^ (29).

For exposure assessment, daily PM₂.₅ concentrations were aggregated into province-level monthly average concentrations for January, February, March, and April 2025. Each participant was assigned the monthly average PM₂.₅ concentration corresponding to his or her province of residence and the month in which the interview was conducted, thereby aligning the exposure assessment with the one-month symptom recall period. Province-level monthly averages were used because the interview survey collected participants’ province of residence rather than precise residential coordinates. Furthermore, monthly averages were considered to better represent cumulative ambient exposure during the recall period while reducing the influence of short-term day-to-day fluctuations.

### 2.4 Statistical analysis

Descriptive statistics were provided by frequency and percentage for categorical variables. Arithmetic mean and standard deviation were used for numerical data with normal distribution. Moreover, geometric mean and standard deviation were reported in data with log-normal distribution, particularly the PM_2.5_ level. Additionally, the median, along with the first and third quartile, was demonstrated alongside for further elaboration. Illustrative choropleth maps were provided for the visualization of spatial distribution of participant number and exposure to PM_2.5_ across the provinces.

Inferential statistics were reported in statistical tests for univariable analysis in relation to presence of symptoms in each health system. Chi-square test was used for categorical determinants; independent sample t-test was used in normally distributed numerical determinants; rank-sum test was carried out for non-normally distributed numerical determinants. Latent class analysis was used to identify protective behavioral phenotypes based on participants’ item-level responses to the protective behavior questions. Models with different numbers of latent classes were compared using the log-likelihood, Akaike information criterion (AIC), and Bayesian information criterion (BIC), as presented in Supplementary Table S2. A gain in log-likelihood of <5% with the addition of another class was considered as an additional stopping criterion for class selection. Class-specific response probabilities are presented in Supplementary Table S3.

Multivariable analysis involved mixed-effect, random-intercept, fixed-slope, binary logistic regression, to account for the potential interviewer variability and spatial clustering of data across 72 provinces as the random intercepts of baseline odds. Intraclass correlation (ICC) was demonstrated to assess the ability of clustering in explaining variance across clusters. Covariates were selected using a combination of prior epidemiological evidence, biological plausibility, conceptual considerations, and findings from univariable analyses, including gender, age, monthly income bracket, education level, co-morbidity, smoking, alcohol use, status as village health volunteer, routine health checkup practice, health behavior phenotype, and PM_2.5_ exposure. Health behavior phenotype was classified according to the latent class analysis into 3 categories based on pattern of the item-wise participants’ responses. PM₂.₅ concentrations were adjusted odds ratios were reported per IQR (15.1 μg/m^³^) increase. Adjusted odds ratio (Adj. OR) and 95% confidence interval (95%CI) were presented in the multivariable analysis.

Model diagnostics were performed for all mixed-effects logistic regression models. Multicollinearity among covariates was assessed using variance inflation factors (VIFs) (Supplementary Table S4). Anscombe residuals were examined to identify potentially influential or outlying observations (Supplementary Figure S1), and the distributions of province-level random intercepts were visually inspected for approximate normality (Supplementary Figures S2, S3). Statistical significance was defined as a two-sided *p*-value <0.05.

### 2.5 Ethics approval

This study was conducted with approval from the Research Ethics Committee of the Faculty of Medicine, Chiang Mai University, Thailand (Approval No.225/2024, approved on 4 June 2024).

## 3 Results

### 3.1 Socio-demographic characteristics of participants

A total of 3,916 participants were included, with a mean age of 41.0 ± 15.4 years and a predominance of female respondents (65.2%). Over half (53.4%) reported a monthly income ≤450 USD, while only 6.3% earned more than 1,500 USD. Nearly half held a bachelor’s degree (45.7%), followed by secondary education (28.0%). Regarding health profiles, 22.1% reported at least one comorbidity, 11.2% were active smokers, and 32.2% consumed alcohol. Health engagement indicators showed that 23.5% served as village health volunteers and 70.7% had undergone routine health checkups.

Participants resided across diverse settings, with 45.5% living in rural areas, 30.6% in suburban, and 24.0% in urban areas. Most participants used cloth or surgical masks (81.8%), while only 18.2% reported using N95 respirators. Exposure assessment indicated that the mean monthly PM₂.₅ concentration was 34.6 ± 12.2 μg/m^3^, substantially exceeding WHO guideline values (Table 1).

**Table 1:** Baseline characteristic of the participants (*n* = 3,916).

Parameters	Categories	*n* (%) or mean ± SD
Gender, *n* (%)	Male	1,361 (34.8)
	Female	2,555 (65.2)
Age (years), mean ± SD		41.0 ± 15.4
Monthly income bracket, *n* (%)	≤450 USD	2,090 (53.4)
	451–1,000 USD	1,158 (29.6)
	1,001–1,500 USD	420 (10.7)
	>1,500 USD	248 (6.3)
Education level, *n* (%)	None	65 (1.7)
	Primary education	664 (17.0)
	Secondary education	1,098 (28.0)
	Bachelor’s degree	1,790 (45.7)
	Postgraduate degree	299 (7.6)
Co-morbidity, *n* (%)		867 (22.1)
Smoking, *n* (%)		437 (11.2)
Alcohol consumption, *n* (%)		1,261 (32.2)
Village health volunteer, *n* (%)		920 (23.5)
Routine health checkup, *n* (%)		2,769 (70.7)
Region of residential area, *n* (%)	Central	1,421 (36.3)
	North	679 (17.3)
	North-East	1,324 (33.8)
	South	492 (12.6)
Residential area, *n* (%)	Rural	1,780 (45.5)
	Suburban	1,198 (30.6)
	Urban	938 (24.0)
Mask type use, *n* (%)	Cloth or surgical	3,203 (81.8)
	N95	713 (18.2)
Monthly PM_2.5_ levels (μg/m^3^)	Mean ± SD	34.6 ± 12.2
	Median (P^25th^, P^75th^)	37.7 (26.3, 41.4)
	Geometric mean	32.0

SD = standard deviation; P^25th^ = the 25th percentile, P^75th^ = the 75th percentile.

### 3.2 Health behaviors during haze season and health behavior phenotypes

During the haze season, participants exhibited high adherence to several low-cost, easily adoptable protective behaviors. The three practices most frequently reported as “always” performed were cleaning indoor areas frequently (64.1%), avoiding smoking outdoors (64.1%), and avoiding smoking indoors (64.0%). In contrast, the three behaviors least frequently performed were using public transportation (17.6%), wearing a mask indoors (17.6%), and using electric vehicles (13.9%) (Supplementary Table S5).

Latent class analysis identified three distinct behavioral phenotypes related to haze-protection practices, including complier, non-complier, and neutral groups. The three-class model was selected for latent class analysis due to the minimal (<5%) gain in log-likelihood ratio in the four and five class models. The complier phenotype (*n* = 1,306) demonstrated consistently high adherence across nearly all behaviors, with probabilities exceeding 85–95% for “always” responses on key practices such as avoiding smoking outdoors (93.2%), avoiding smoking indoors (92.2%), cleaning indoor areas (91.1%), wearing a mask outdoors (86.1%), and avoiding open waste burning (86.1%). This group also showed strong adoption of other protective behaviors, including keeping windows closed (84.9%) and turning off the car engine when parked (75.4%). Although adherence was slightly lower for higher-cost or technology-dependent practices (e.g., using a home air purifier or electric vehicle), compliers still maintained substantially higher adherence than other groups. In contrast, the non-complier phenotype (*n* = 1,905) displayed markedly lower engagement in protective behaviors, with many “always” responses falling below 15%, including postponing travel to polluted areas (12.5%), working from home during haze season (9.6%), avoiding grilling food (12.8%), using a home air purifier (9.9%), wearing a mask indoors (4.7%), using a car air purifier (6.7%), using a bicycle for transportation (9.6%), using public transportation (6.4%), and using an electric vehicle (3.9%). Their adherence was generally distributed between “never” and “sometimes,” indicating inconsistent protection. The neutral phenotype (*n* = 705) was characterized by overwhelmingly predominant “sometimes” responses (ranging from 77.7 to 95.1%), reflecting moderate yet inconsistent adherence. Most protective behaviors in this group showed low probabilities of “always” responses (generally <10%), indicating limited adoption of strong protective measures (Table 2).

**Table 2:** Latent class analysis classifying the three major behavioral phenotypes; each cell contains the probability of answering a 3-scale, frequency-based self-responses questionnaire about haze season practices.

Response frequency
Items	Phenotype 1 complier (*n* = 1,306)			Phenotype 2 non-complier (*n* = 1,905)			Phenotype 3 neutral (*n* = 705)		
	Never	Sometimes	Always	Never	Sometimes	Always	Never	Sometimes	Always
Avoid smoking outdoors	2.0%	4.8%	93.2%	28.3%	6.8%	64.8%	9.4%	82.3%	8.3%
Avoid smoking indoors	2.1%	5.6%	92.2%	28.8%	5.9%	65.3%	9.5%	82.2%	8.2%
Clean indoor areas frequently	0.2%	8.8%	91.1%	5.3%	27.5%	67.2%	1.1%	92.7%	6.1%
Avoid open waste burning	2.0%	12.0%	86.1%	20.1%	27.5%	52.4%	4.3%	87.7%	8.0%
Wear a mask outdoors	0.2%	13.7%	86.1%	5.4%	58.2%	36.4%	1.5%	90.1%	8.4%
Keep windows closed	1.0%	14.1%	84.9%	13.7%	45.3%	40.9%	4.9%	89.7%	5.4%
Turn off the car engine when parked	3.4%	21.2%	75.4%	17.9%	32.6%	49.5%	1.3%	90.8%	7.9%
Buy certified pollution-free products	1.6%	23.6%	74.8%	29.5%	45.3%	25.2%	3.1%	94.2%	2.7%
Avoid outdoor activities	0.7%	25.5%	73.7%	15.8%	61.4%	22.7%	1.9%	95.1%	2.9%
Use an electric stove instead of coal	4.5%	22.1%	73.4%	21.2%	33.9%	45.0%	1.0%	94.9%	4.2%
Postpone travel to polluted areas	6.2%	34.2%	59.5%	32.0%	55.5%	12.5%	1.8%	93.7%	4.5%
Work from home during haze season	16.4%	30.6%	53.0%	59.9%	30.5%	9.6%	12.1%	86.1%	1.8%
Avoid grilling food	5.0%	44.3%	50.7%	31.4%	55.8%	12.8%	3.5%	95.1%	1.5%
Use a home air purifier	27.9%	22.4%	49.7%	73.5%	16.5%	9.9%	18.5%	77.7%	3.8%
Wear a mask indoors	17.5%	38.5%	44.0%	64.3%	30.9%	4.7%	13.3%	83.6%	3.0%
Use a car air purifier	31.3%	25.5%	43.2%	78.0%	15.3%	6.7%	18.0%	78.0%	4.0%
Use a bicycle for transportation	15.7%	44.0%	40.3%	51.0%	39.4%	9.6%	4.4%	90.9%	4.7%
Use public transportation	19.9%	40.1%	40.0%	58.7%	35.0%	6.4%	4.3%	89.7%	6.0%
Use an electric vehicle	44.8%	21.6%	33.7%	84.7%	11.5%	3.9%	10.9%	85.4%	3.8%

The different color are used solely for visual distinction among the 3 phenotypes.

Table 3 presents sociodemographic and behavioral distinctions across the three behavioral phenotypes. All sociodemographic parameters, except monthly income, differed significantly among the groups. Compliers were more likely to be female (72.7%), older (mean age 42.6 years), and to have at least one comorbidity (25.3%). They also demonstrated the highest levels of health engagement, evidenced by the greatest prevalence of routine health checkups (77.6%) and village health volunteer participation (31.0%). Additionally, Compliers reported the lowest rates of smoking (6.4%) and alcohol consumption (24.5%). In contrast, Non-compliers were younger on average (40.6 years), had higher rates of smoking (11.9%) and alcohol consumption (36.9%), and showed the lowest participation in both routine health checkups and village health volunteer activities, suggesting low engagement with personal and community health practices. The Neutral phenotype, which accounted for nearly one-fifth of the sample, had the youngest mean age (39.4 years), a more balanced gender distribution, and the highest smoking prevalence (18.0%). This group also exhibited the lowest levels of comorbidity (15.3%) and lower educational attainment, with a larger proportion reporting only secondary education. Monthly PM₂.₅ exposure was generally comparable across phenotypes, although slight differences were observed in median values (*p*-value = 0.006).

**Table 3:** Baseline characteristic of the participants classified by behavioral phenotypes (*n* = 3,916).

Determinants	Categories	Total	Phenotype 1 Complier (n = 1,306)	Phenotype 2 Non-complier (n = 1,905)	Phenotype 3 Neutral (n = 705)	*p*-value
Gender	Male	1,361 (34.8)	356 (27.3)	662 (34.8)	343 (48.7)	<0.001**
	Female	2,555 (65.2)	950 (72.7)	1,243 (65.2)	362 (51.3)	
Age (years), mean ± SD		41.0 ± 15.4	42.6 ± 16.0	40.6 ± 15.2	39.4 ± 14.8	<0.001**
Monthly income bracket	≤450 USD	2,090 (53.4)	696 (53.3)	1,002 (52.6)	392 (55.6)	0.16
	451–1,000 USD	1,158 (29.6)	364 (27.9)	591 (31.0)	203 (28.8)	
	1,001–1,500 USD	420 (10.7)	149 (11.4)	196 (10.3)	75 (10.6)	
	>1,500 USD	248 (6.3)	97 (7.4)	116 (6.1)	35 (5.0)	
Education level	None	65 (1.7)	18 (1.4)	41 (2.2)	6 (0.9)	<0.001**
	Primary education	664 (17.0)	218 (16.7)	306 (16.1)	140 (19.9)	
	Secondary education	1,098 (28.0)	359 (27.5)	499 (26.2)	240 (34.0)	
	Bachelor’s degree	1,790 (45.7)	592 (45.3)	914 (48.0)	284 (40.3)	
	Postgraduate degree	299 (7.6)	119 (9.1)	145 (7.6)	35 (5.0)	
Co-morbidity		867 (22.1)	330 (25.3)	429 (22.5)	108 (15.3)	<0.001**
Smoking		437 (11.2)	84 (6.4)	226 (11.9)	127 (18.0)	<0.001**
Alcohol consumption		1,261 (32.2)	320 (24.5)	702 (36.9)	239 (33.9)	<0.001**
Village health volunteer		920 (23.5)	405 (31.0)	321 (16.9)	194 (27.5)	<0.001**
Routine health checkup		2,769 (70.7)	1,013 (77.6)	1,263 (66.3)	493 (69.9)	<0.001**
Residential area	Rural	1,780 (45.5)	469 (35.9)	962 (50.5)	349 (49.5)	
	Suburban	1,198 (30.6)	466 (35.7)	512 (26.9)	220 (31.2)	<0.001**
	Urban	938 (24.0)	371 (28.4)	431 (22.6)	136 (19.3)	
Mask type	Cloth or surgical	3,203 (81.8)	980 (75.0)	1,617 (84.9)	606 (86.0)	<0.001**
	N95	713 (18.2)	326 (25.0)	288 (15.1)	99 (14.0)	
Monthly PM_2.5_ (μg/m^3^)	Mean±SD	34.6 ± 12.2	34.5 ± 11.5	34.9 ± 12.6	34.0 ± 12.5	0.29
	Median (P^25th^, P^75th^)	37.7 (26.3, 41.4)	36.6 (26.2, 40.8)	37.9 (26.3, 41.9)	37.7 (24.8, 40.3)	0.006**
	Geometric mean	32.0	32.3	32.1	31.2	0.20

SD = standard deviation; P^25th^ = the 25th percentile, P^75th^ = the 75th percentile; ** *p*-value <0.01.

### 3.3 Spatial distribution of monthly average PM₂.₅ levels across Thailand from January to April 2025

Figure 2 illustrates the spatial distribution and temporal variation of monthly average PM₂.₅ concentrations across Thailand from January to April 2025. In January 2025, elevated PM₂.₅ levels were observed nationwide, with the highest concentrations in the Northern and Central regions, while the Southern region exhibited comparatively lower levels. PM₂.₅ concentrations increased further in February 2025, particularly across the Northern, Northeastern, and Central regions, indicating a widespread intensification of particulate pollution compared with January. In March 2025, PM₂.₅ levels remained persistently high in the Northern and Northeastern regions, with only slight improvement in Central Thailand, suggesting sustained atmospheric accumulation. By April 2025, a general decline in PM₂.₅ levels was observed, especially in the Central and Southern regions; however, several Northern provinces continued to experience concentrations above recommended air quality thresholds. Overall, the January–April 2025 average PM₂.₅ distribution demonstrates consistently the highest pollution burdens in Northern Thailand, followed by the Northeastern and Central regions, compared with the Southern region, reflecting pronounced seasonal and spatial heterogeneity in ambient air pollution during the haze period.

**Figure 2:**
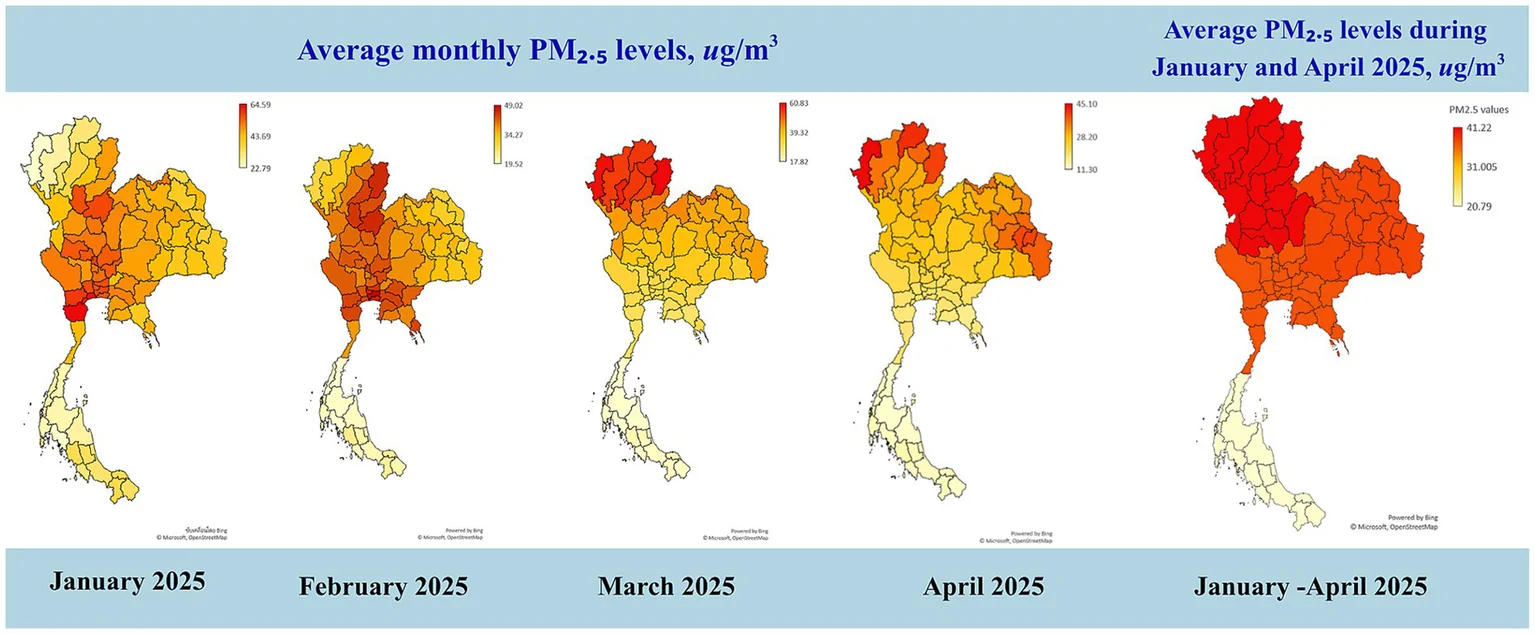
Spatial distribution of monthly average PM_2.5_ levels during haze season across Thailand (January to April 2025).

### 3.4 Acute health symptoms during haze season

The prevalence of acute symptoms during the haze season is shown in Figure 3. Respiratory symptoms were the most prevalent (80.7%), with common manifestations including coughing, nasal congestion, and runny nose, reflecting upper airway irritation from particulate exposure. Eye symptoms (52.8%) were also frequently reported, predominantly eye itchiness and burning eyes. Skin symptoms (48.7%), such as acne and white/red rashes, were similarly widespread. Neurological symptoms (33.1%), including headache, and migraine, were reported by approximately one-third of participants.

**Figure 3:**
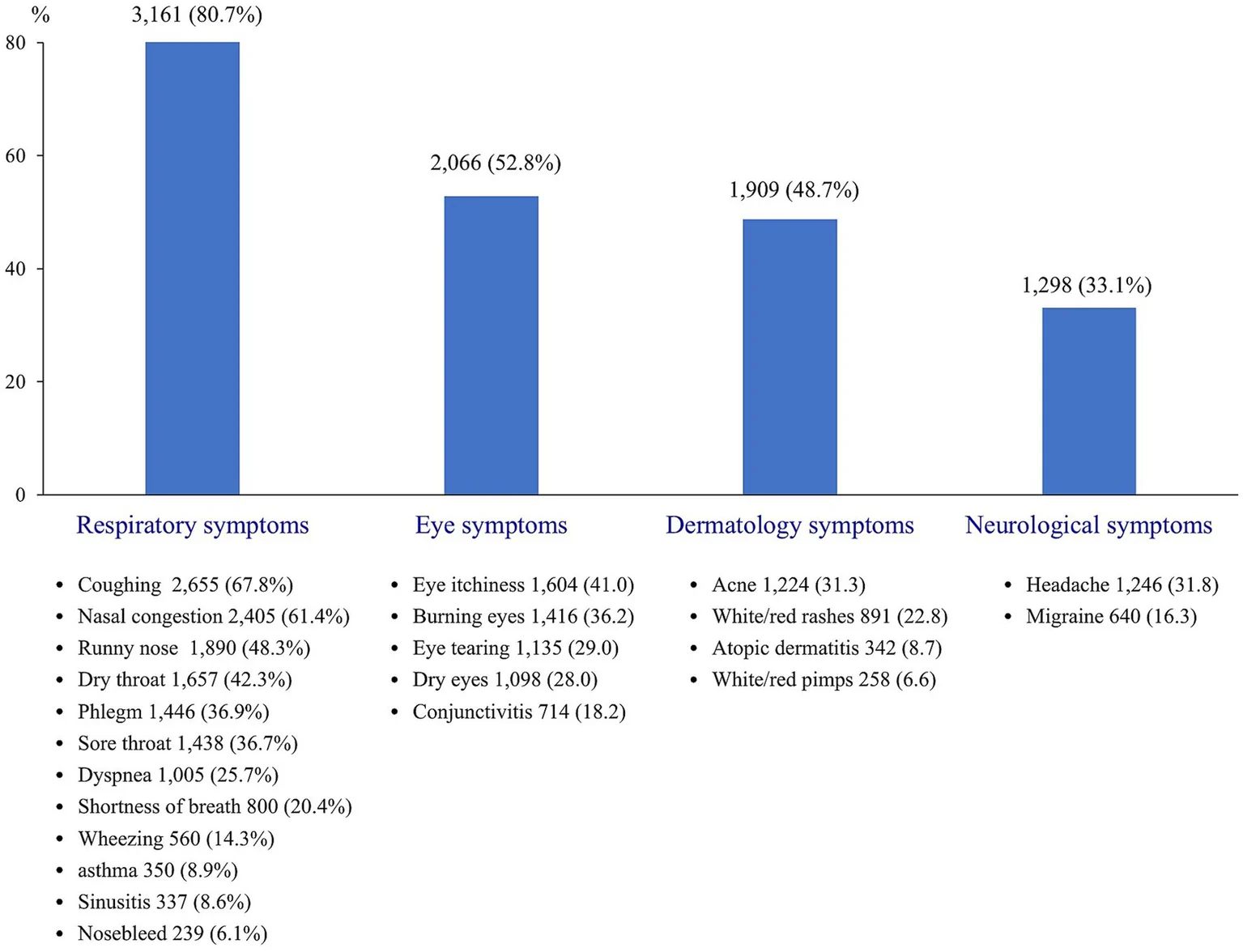
Acute health symptoms during haze season in Thailand.

Across all acute symptom categories, significant sociodemographic differences emerged between participants who reported symptoms and those who did not. Females consistently exhibited higher prevalence of respiratory, eye, skin, and neurological symptoms compared with males (all *p*-value < 0.01). Participants with symptoms were also younger, with mean ages approximately 2–8 years lower than those without symptoms. Education levels were associated with all symptom types (all *p*-value < 0.01). the presence of comorbidity was linked to increased respiratory, eye, and skin symptoms. Smoking was more prevalent among participants with skin and neurological symptoms, whereas alcohol consumption was higher among those reporting respiratory, eye and skin symptoms.

Behavioral and Environmental factors also varied significantly by symptom status. Participants with symptoms were slightly more likely to reside in rural areas, and they were significantly less likely to be village health volunteers. Notably, village health volunteers consistently exhibited a lower prevalence of all symptom categories (all *p* value < 0.01), suggesting a protective role associated with greater health awareness or preventive practices. Symptomatic individuals were more likely to use cloth or surgical masks rather than N95 masks. Behavioral phenotypes also differed. Compliers were more frequent among those without symptoms, whereas non-compliers were more prevalent among symptomatic individuals. Importantly, monthly PM₂.₅ concentrations were significantly higher among participants reporting respiratory, eye, and skin symptoms, reflected in higher means, medians, and geometric means (all *p* value < 0.01) (Table 4).

**Table 4:** Baseline characteristic of the participants classified by acute health symptoms during haze season (*n* = 3,916).

Determinants	Total (*n* = 3,916)	Respiratory symptoms			Eye symptoms			Skin symptoms			Neurological symptoms		
		Yes (*n* = 3,161)	No (*n* = 755)	Sig.	Yes (*n* = 2,066)	No (*n* = 1,850)	Sig.	Yes (*n* = 1,909)	No (*n* = 2,007)	Sig.	Yes (*n* = 1,298)	No (*n* = 2,618)	Sig.
Gender													
Male	1,361 (34.8)	1,072 (33.9)	289 (38.3)	*	673 (32.6)	688 (37.2)	**	564 (29.5)	797 (39.7)	**	355 (27.3)	1,006 (38.4)	**
Female	2,555 (65.2)	2,089 (66.1)	466 (61.7)		1,393 (67.4)	1,162 (62.8)		1,345 (70.5)	1,210 (60.3)		943 (72.7)	1,612 (61.6)	
Age (years), mean ± SD	41.0 ± 15.4	40.2 ± 15.0	44.6 ± 16.7	**	39.5 ± 14.9	42.8 ± 15.8	**	36.6 ± 13.9	45.3 ± 15.7	**	37.9 ± 14.3	42.6 ± 15.8	**
Monthly income bracket													
≤450 USD	2,090 (53.4)	1,661 (52.5)	429 (56.8)	ns	1,085 (52.5)	1,005 (54.3)	ns	1,007 (52.8)	1,083 (54.0)	ns	730 (56.2)	1,360 (51.9)	**
451–1,000 USD	1,158 (29.6)	949 (30.0)	209 (27.7)		617 (29.9)	541 (29.2)		590 (30.9)	568 (28.3)		386 (29.7)	772 (29.5)	
1,001–1,500 USD	420 (10.7)	351 (11.1)	69 (9.1)		216 (10.5)	204 (11.0)		186 (9.7)	234 (11.7)		110 (8.5)	310 (11.8)	
>1,500 USD	248 (6.3)	200 (6.3)	48 (6.4)		148 (7.2%)	100 (5.4)		126 (6.6)	122 (6.1)		72 (5.5)	176 (6.7)	
Education level													
None	65 (1.7)	56 (1.8)	9 (1.2)	**	47 (2.3)	18 (1.0)	**	28 (1.5)	37 (1.8)	**	24 (1.8)	41 (1.6)	**
Primary education	664 (17.0)	493 (15.6)	171 (22.6)		246 (11.9)	418 (22.6)		158 (8.3)	506 (25.2)		147 (11.3)	517 (19.7)	
Secondary education	1,098 (28.0)	848 (26.8)	250 (33.1)		546 (26.4)	552 (29.8)		478 (25.0)	620 (30.9)		335 (25.8)	763 (29.1)	
Bachelor’s degree	1,790 (45.7)	1,512 (47.8)	278 (36.8)		1,040 (50.3)	750 (40.5)		1,065 (55.8)	725 (36.1)		674 (51.9)	1,116 (42.6)	
Postgraduate degree	299 (7.6)	252 (8.0)	47 (6.2)		187 (9.1)	112 (6.1)		180 (9.4)	119 (5.9)		118 (9.1)	181 (6.9)	
Co-morbidity	867 (22.1)	721 (22.8)	146 (19.3)	*	503 (24.3)	364 (19.7)	**	396 (20.7)	471 (23.5)	*	285 (22.0)	582 (22.2)	ns
Smoking	437 (11.2)	350 (11.1)	87 (11.5)	ns	218 (10.6)	219 (11.8)	ns	155 (8.1)	282 (14.1)	**	110 (8.5)	327 (12.5)	**
Alcohol consumption	1,261 (32.2)	1,044 (33.0)	217 (28.7)	*	750 (36.3)	511 (27.6)	**	651 (34.1)	610 (30.4)	*	423 (32.6)	838 (32.0)	ns
Village health volunteer	920 (23.5)	664 (21.0)	256 (33.9)	**	380 (18.4)	540 (29.2)	**	322 (16.9)	598 (29.8)	**	244 (18.8)	676 (25.8)	**
Routine health checkup	2,769 (70.7)	2,258 (71.4)	511 (67.7)	*	1,485 (71.9)	1,284 (69.4)	ns	1,345 (70.5)	1,424 (71.0)	ns	914 (70.4)	1,855 (70.9)	ns
Residential area													
Rural	1,780 (45.5)	1,415 (44.8)	365 (48.3)	ns	885 (42.8)	895 (48.4)	**	781 (40.9)	999 (49.8)	**	565 (43.5)	1,215 (46.4)	*
Suburban	1,198 (30.6)	970 (30.7)	228 (30.2)		616 (29.8)	582 (31.5)		573 (30.0)	625 (31.1)		386 (29.7)	812 (31.0)	
Urban	938 (24.0)	776 (24.5)	162 (21.5)		565 (27.3)	373 (20.2)		555 (29.1)	383 (19.1)		347 (26.7)	591 (22.6)	
Mask type													
Cloth or surgical	3,203 (81.8)	2,567 (81.2)	636 (84.2)	ns	1,640 (79.4)	1,563 (84.5)	**	1,495 (78.3)	1,708 (85.1)	**	1,036 (79.8)	2,167 (82.8)	*
N95	713 (18.2)	594 (18.8)	119 (15.8)		426 (20.6)	287 (15.5)		414 (21.7)	299 (14.9)		262 (20.2)	451 (17.2)	
Behavior phenotype													
Complier	1,306 (33.4)	972 (30.7)	334 (44.2)	**	667 (32.3)	639 (34.5)	**	633 (33.2)	673 (33.5)	**	407 (31.4)	899 (34.3)	*
Non-complier	1,905 (48.6)	1,606 (50.8)	299 (39.6)		1,093 (52.9)	812 (43.9)		985 (51.6)	920 (45.8)		673 (51.8)	1,232 (47.1)	
Neutral	705 (18.0)	583 (18.4)	122 (16.2)		306 (14.8)	399 (21.6)		291 (15.2)	414 (20.6)		218 (16.8)	487 (18.6)	
Monthly PM_2.5_ (μg/m^3^)													
Mean ± SD	34.6 ± 12.2	35.2 ± 12.1	31.9 ± 12.1	**	35.3 ± 12.2	33.8 ± 12.2	**	35.4 ± 12.7	33.9 ± 11.7	**	35.1 ± 12.5	34.4 ± 12.1	ns
Median (P^25th^, P^75th^)	37.7 (26.3, 41.4)	37.7 (26.3, 41.7)	35.0 (23.3, 40.4)	**	37.8 (26.3, 41.7)	36.6 (24.8, 41.4)	**	37.7 (26.3, 42.6)	37.5 (26.2, 40.8)	**	37.7 (26.3, 41.8)	37.6 (26.3, 41.4)	ns
Geometric mean	32.0	32.7	29.3	**	32.7	31.2	**	32.6	31.4	**	32.5	31.8	ns

Sig. = significance; ns = non-significance; SD = standard deviation; P^25th^ = the 25^th^ percentile, P^75th^ = the 75^th^ percentile; *p-value < 0.05; **p-value < 0.01.

### 3.5 Factors associated with acute health symptoms during haze season

Table 5 presents the mixed-effects multivariable binary logistic regression examining factors associated with acute health symptoms during the haze season. Female gender was consistently associated with higher odds of all symptom types, including respiratory (adj.OR = 1.42, 95%CI = 1.15, 1.75), eye (adj.OR = 1.35, 95%CI = 1.14, 1.60), skin (adj.OR = 1.52, 95%CI = 1.28, 1.81), and neurological symptoms (adj.OR = 1.73, 95%CI = 1.45, 2.07). Younger age was also a significant predictor; every 10-year increase in age reduced the odds of symptoms by 9–30% depending on the symptom category. The presence of comorbidity substantially increased the likelihood of respiratory (adj.OR = 1.94, 95%CI = 1.50, 2.51), eye (adj.OR = 1.73, 95%CI = 1.42, 2.11), skin (adj.OR = 1.75, 95%CI = 1.44, 2.14), and neurological symptoms (adj.OR = 1.36, 95%CI = 1.12, 1.66). Higher educational attainment also increased the odds of eye (adj.OR = 1.20, 95%CI = 1.06, 1.34), skin (adj.OR = 1.26, 95%CI = 1.11, 1.41), and neurological symptoms (adj.OR = 1.16, 95%CI = 1.03, 1.31). Alcohol consumption was associated with greater odds of eye (adj.OR = 1.45, 95%CI = 1.22, 1.72) and skin symptoms (adj.OR = 1.24, 95%CI = 1.04, 1.48). Notably, village health volunteers consistently demonstrated lower odds across all symptom categories (adj.OR = 0.60 for respiratory, eye, and skin symptoms, and 0.73 for neurological symptoms).

**Table 5:** Mixed-effect multivariable binary logistic regression exploring factors associated with acute health symptoms during haze season.

Factors	Respiratory symptom		Eye symptom		Skin symptom		Neurological symptom	
	Adj. OR	95%CI	Adj. OR	95%CI	Adj. OR	95%CI	Adj. OR	95%CI
Sociodemographic determinants								
Gender (Female vs. Male (ref.))	1.42	1.15, 1.75**	1.35	1.14, 1.60**	1.52	1.28, 1.81**	1.73	1.45, 2.07**
Age (every 10-year increase)	0.83	0.76, 0.90**	0.91	0.85, 0.98**	0.70	0.66, 0.75**	0.86	0.81, 0.93**
Monthly income bracket (every 1-order increase)	0.93	0.82, 1.06	0.92	0.83, 1.02	0.91	0.82, 1.01	0.84	0.75, 0.93**
Education level (every 1-order increase)	1.04	0.90, 1.21	1.20	1.06, 1.34**	1.26	1.11, 1.41**	1.16	1.03, 1.31*
Co-morbidity (Yes vs. No.(ref.))	1.94	1.50, 2.51**	1.73	1.42, 2.11**	1.75	1.44, 2.14**	1.36	1.12, 1.66**
Lifestyle and protective behavior determinants								
Smoking (Yes vs. No.(ref.))	0.85	0.61, 1.17	0.96	0.74, 1.24	0.74	0.57, 0.97*	0.96	0.73, 1.27
Alcohol consumption (Yes vs. No.(ref.))	1.10	0.87, 1.37	1.45	1.22, 1.72**	1.24	1.04, 1.48*	1.05	0.89, 1.25
Village health volunteer (Yes vs. No.(ref.))	0.60	0.47, 0.77**	0.60	0.49, 0.74**	0.60	0.49, 0.74**	0.73	0.59, 0.91**
Routine health checkup (Yes vs. No.(ref.))	1.18	0.95, 1.48	1.04	0.87, 1.24	1.14	0.96, 1.37	1.10	0.93, 1.32
Mask type use (N95 vs. Cloth or surgical as ref.)	1.04	0.81, 1.33	0.97	0.81, 1.17	0.88	0.73, 1.07	0.99	0.82, 1.20
Behavior classification (Complier as ref.)								
Non-complier	1.71	1.38, 2.13**	1.15	0.97, 1.37	1.18	0.99, 1.40	1.31	1.10, 1.56**
Neutral	1.65	1.25, 2.18**	0.85	0.68, 1.07	0.91	0.73, 1.15	1.32	1.05, 1.66*
Environmental determinants								
Residential area (Rural as ref.)								
Suburban	1.49	1.16, 1.89**	1.27	1.06, 1.53*	1.15	0.95, 1.39	1.12	0.93, 1.35
Urban	1.20	0.91, 1.57	1.11	0.90, 1.37	1.40	1.13, 1.74**	1.13	0.91, 1.40
Monthly PM_2.5_ levels (every IQR or 15.1 μg/m^3^ increase)	1.02	1.01, 1.04**	1.01	1.00, 1.02	1.01	1.00,1.02*	1.01	1.00, 1.02
Intraclass correlation (ICC), 95%CI	0.22 (0.15, 0.30)		0.17 (0.12, 0.24)		0.14 (0.09, 0.20)		0.10 (0.07, 0.16)	

*p-value < 0.05; **p-value < 0.01.

Environmental and behavioral determinants also contributed significantly to symptom risks. Living in suburban areas increased the likelihood of respiratory (adj.OR = 1.49, 95%CI = 1.16, 1.89) and eye symptoms (adj.OR = 1.27, 95%CI = 1.06, 1.53), while urban residence was significantly associated with skin symptoms (adj.OR = 1.40, 95%CI = 1.13, 1.74). Behavioral phenotypes emerged as important predictors. When comparing with compliers, non-compliers had higher odds of respiratory (adj.OR = 1.71, 95%CI = 1.38, 2.13) and neurological symptoms (adj. OR = 1.31, 95%CI = 1.10, 1.56), and neutral participants displayed similar elevations in respiratory (adj.OR = 1.65, 95%CI = 1.25, 2.18) and neurological symptoms (adj.OR = 1.32, 95%CI = 1.05, 1.66). Exposure to higher PM₂.₅ levels remained a strong independent predictor, with each IQR (15.1 μg/m³) increase in PM₂.₅ associated with higher odds of respiratory symptoms (adj.OR = 1.02, 95%CI = 1.01, 1.04) and significantly increasing skin symptoms (adj.OR = 1.01, 95%CI = 1.00, 1.02). Random-intercept ICC values ranging from 0.10–0.22 indicated meaningful provincial-level clustering of health outcomes.

Exploratory analyses were conducted to assess potential effect modification between protective behavioral phenotypes and PM₂.₅ exposure for each health outcome. Significant interactions were observed for respiratory (*p* = 0.013) and dermatological symptoms (*p* = 0.031), whereas no significant interactions were observed for eye (*p* = 0.338) or neurological symptoms (*p* = 0.187). Model diagnostics were subsequently performed for all mixed-effects logistic regression models. No serious multicollinearity was detected, with a mean variance inflation factor (VIF) of 1.34 and no VIF values exceeding 10. Examination of Anscombe residuals indicated only minimal outlying observations in the respiratory symptom model and no evident outliers in the other models. Visual inspection of the random-intercept distributions indicated approximate normality across all models. In addition, the estimated intraclass correlation coefficients (ICCs) exceeded 10% in all models, indicating meaningful clustering at the provincial level and supporting the use of random-intercept mixed-effects models rather than conventional logistic regression models that assume independence of observations. The sensitivity analysis of each mixed-effect logistic regression models with PM_2.5_ specification as linear and restricted cubic splines terms demonstrated by log-likelihood terms and tests of log-likelihood ratios (Supplementary Table S6).

## 4 Discussion

This study presents comprehensive evidence on haze-related protective behaviors among the Thai population and identifies distinct behavioral phenotypes during haze season. Although PM₂.₅ levels during this period exceeded WHO guideline values, individuals exhibited substantial heterogeneity in protective practices, underscoring important behavioral and sociodemographic differences in haze-related health responses. In our study, participants showed higher adherence to low-cost, easily implementable protective behaviors, such as indoor cleaning, avoiding smoking, and avoiding open waste burning, whereas behaviors requiring greater financial investment, technological reliance, or supporting infrastructure, including the use of air purifiers, electric vehicles, and public transportation, were markedly less prevalent. These findings align with prior evidence suggesting that individuals are more likely to adopt behaviors requiring minimal financial investment or structural change, particularly in resource-constrained settings (20–22). The evidence further suggested that higher health risk perception, positive attitudes, and supportive subjective norms are associated with increased engagement in protective measures, whereas perceived barriers, such as cost, convenience, and limited access to technology, reduce the adoption of higher-cost or infrastructure-dependent behaviors (20, 21). Consequently, low-cost and easily implementable actions (e.g., mask use, window closure, and reduced outdoor exposure) are more commonly practiced, underscoring the critical role of structural feasibility in shaping individual responses to air pollution. A previous study in the Thai population similarly reported that although consumers perceived PM₂.₅ as dangerous and expressed strong negative attitudes toward air pollution, perceived risk alone did not consistently translate into environmentally friendly consumer behavior (22). Thai consumers predominantly adopted familiar, convenient, and low-cost protective behaviors, such as closing windows and wearing masks, but were less willing to modify habitual or culturally embedded practices, such as cooking methods that generate smoke or lighting incense during festivals, highlighting clear behavioral trade-offs between environmental protection and convenience or tradition (22). Moreover, a recent systematic review by Ounsaneha et al. (2026) (23) demonstrated that protective behaviors against PM₂.₅ exposure are strongly predicted by psychosocial factors, particularly risk perception, self-efficacy, and social norms, across diverse exposure contexts. Individuals who perceived higher personal risk, felt confident in their ability to act, and observed protective behaviors as socially normative were significantly more likely to adopt measures such as mask use, activity reduction, and indoor protection, indicating that information alone is insufficient without addressing these psychosocial drivers. Collectively, these findings suggest that structural, cultural, and economic constraints, rather than limited awareness alone, may be associated with differences in protective behavior adoption and highlight the limitations of relying solely on individual-level responsibility to mitigate air pollution exposure. Taken together, the evidence underscores the need for policy interventions that extend beyond individual behavior change and instead focus on reducing structural, cultural, and economic barriers, including improving access to affordable protective technologies, strengthening public infrastructure, and implementing upstream pollution control measures to effectively reduce population-level health risks from air pollution.

Our study identified three distinct protective behavioral phenotypes such as compliers, non-compliers, and neutrals, highlighting substantial heterogeneity in protective responses despite similar ambient PM₂.₅ exposure levels. This finding suggests that differences in symptom burden were associated not only with environmental exposure but also with individual, social, and behavioral factors that influence risk management. Participants in the complier phenotype demonstrated consistently high adherence to protective behaviors and were generally older, had a higher prevalence of comorbidities, and were more actively engaged with formal and informal health services, including routine health checkups and participation as village health volunteers. These characteristics may reflect greater health consciousness, higher perceived susceptibility to air pollution–related health effects, and stronger engagement in preventive practices. At the population level, these findings indicate that exposure to similar ambient PM₂.₅ concentrations does not necessarily result in the same health outcomes. Instead, behavioral heterogeneity may partly explain differences in vulnerability among individuals living under comparable environmental conditions. Consequently, public health strategies should extend beyond monitoring ambient PM₂.₅ concentrations to include interventions that promote protective behaviors, particularly among population subgroups with lower adherence to recommended preventive measures.

Several previous studies claimed that higher perceived susceptibility is generally associated with greater engagement in protective behaviors, as individuals who believe they are personally at risk are more motivated to consider and initiate preventive actions. However, perceived susceptibility alone does not reliably translate into behavior change; its influence is strongest when combined with perceived severity, perceived benefits, perceived barriers, and self-efficacy (30). In addition, individuals who engage in proactive health behaviors tend to exhibit higher levels of risk perception and are more likely to actively seek, access, and utilize health information when adopting protective practices (31). Supporting this framework, Ng et al. (2021) (32) reported that risk perception partially mediates the relationship between knowledge and protective behavior during haze episodes. In the Thai context, village health volunteers play a pivotal role as community-based health intermediaries, supporting health promotion, disease prevention, and health surveillance. Their responsibilities include educating community members, monitoring health conditions, collecting health-related data, addressing basic healthcare needs, and facilitating linkages to formal health services, particularly for vulnerable populations such as older adults (33, 34). Consequently, village health volunteers are likely to possess heightened knowledge, greater risk perception, and enhanced access to health information related to air pollution, positioning them as key agents in promoting protective behaviors against PM₂.₅ exposure.

In contrast, the non-complier phenotype exhibited consistently low adherence to protective behaviors. This group was younger, more likely to smoke and consume alcohol, and less engaged in preventive care, suggesting lower risk perception and clustering of unhealthy lifestyle behaviors that may limit the effectiveness of information-based interventions alone. The neutral phenotype, by comparison, appeared to reflect behavioral ambivalence rather than firm non-adherence. Individuals in this group were younger, had lower educational attainment, fewer comorbidities, and the highest prevalence of smoking, indicating a potentially transitional group that may be particularly responsive to targeted, context-sensitive interventions. Notably, monthly PM₂.₅ exposure levels were comparable across all phenotypes, indicating that differences in protective behaviors were not driven by differential PM_2.5_ exposure. This finding suggests that differences in protective behaviors may contribute to variation in personal PM₂.₅ exposure even among individuals experiencing similar ambient PM₂.₅ concentrations (35).

Overall, our results suggest that identifying distinct behavioral phenotypes provides a valuable framework for designing tailored public health interventions. Universal risk messaging alone may be insufficient to address heterogeneous behavioral responses. Leveraging contact with the health system may provide opportunities to promote protective behaviors among compliers, whereas non-compliers may require integrated interventions that simultaneously address smoking, alcohol use, and risk perception. For the neutral group, simplified communication strategies that enhance behavioral consistency and self-efficacy are particularly warranted. Incorporating these behavioral insights into national haze-mitigation policies and community-based health promotion programs may ultimately strengthen population resilience to recurrent air pollution episodes.

Regarding acute health symptoms during the haze season in Thailand, our findings indicate a substantial burden of multisystem symptoms, with respiratory manifestations being the most prevalent, followed by eye, dermatological, and neurological symptoms. This pattern is consistent with previous evidence that PM₂.₅ exposure affects multiple organ systems through inflammatory and oxidative stress pathways (7–9). Respiratory effects are primarily attributed to oxidative stress, inflammatory responses, and airway injury (7). The high prevalence of eye and dermatological symptoms is also biologically plausible, leading to epithelial damage, immune activation, oxidative stress, barrier dysfunction, and impaired tissue repair (36–39). In addition, the occurrence of neurological symptoms is consistent with evidence that PM₂.₅ may induce neuroinflammation, oxidative stress, mitochondrial dysfunction, blood–brain barrier disruption, and other neurotoxic processes (8, 9, 40).

Importantly, our results indicate that acute health symptoms during the haze season were associated with a combination of sociodemographic, lifestyle-related, and environmental factors. Female sex was consistently associated with higher odds of respiratory, eye, dermatological, and neurological symptoms. This finding is consistent with previous epidemiological studies reporting greater susceptibility among women during air pollution episodes, potentially reflecting a combination of biological and social factors. Biological mechanisms include sex-specific hormonal and immune regulation, differences in airway anatomy and lung physiology, and greater susceptibility to neurological disorders such as migraine (41–44). In addition, gender-related differences in household air pollution exposure, socioeconomic disadvantage, and adaptive capacity may further contribute to the higher symptom burden observed among women, particularly in low- and middle-income settings (45).

Our findings also demonstrated a negative association between age and the prevalence of all symptom categories, likely that younger individuals may experience greater exposure through occupational demands and outdoor activities, resulting in higher air pollution exposure (46, 47). Contrast, older adults often spend more time indoors and may reduce outdoor activities during periods of poor air quality, thereby lowering inhaled pollutants doses (48, 49). Older adults may also perceive air pollution as a serious health threat and are more likely to adopt protective actions, which can further reduce short-term symptom occurrence (50, 51). The presence of comorbid conditions also substantially increased the likelihood of all acute symptom categories, underscoring the heightened vulnerability of individuals with underlying health conditions to particulate matter exposure (52). These findings reinforce longstanding public health concerns regarding the disproportionate health impacts of air pollution among medically and socially vulnerable populations.

Lifestyle behaviors were also associated with air pollution related health symptoms. In our study, alcohol consumption was associated with increased odds of eye and dermatological symptoms, potentially through alcohol-induced oxidative stress, inflammation, and immune modulation, which may exacerbate mucosal and dermal irritation (53–55). Our findings further indicate that village health volunteers consistently exhibited lower odds of all symptom categories, suggesting that higher health literacy, preventive behaviors, and risk awareness may be associated with lower odds of symptoms. In Thailand, village health volunteers serve as important health information intermediaries within their communities, reinforcing protective norms and collective risk awareness (33, 34). Notably, the identification of distinct behavioral phenotypes further emphasizes the critical role of consistent and comprehensive protective practices. Individuals classified as non-compliers or belonging to the neutral phenotype exhibited significantly higher odds of respiratory and neurological symptoms compared with compliers, even after adjustment for PM₂.₅ exposure and individual characteristics. This pattern suggests that partial or inconsistent adoption of protective behaviors was associated with higher odds of respiratory and neurological symptoms, although causal relationships cannot be inferred. Respiratory and neurological symptoms may be particularly sensitive to the combined effects of particulate matter exposure and inadequate mitigation, potentially mediated through systemic inflammation, oxidative stress, immune dysregulation, and epigenetic modifications (9, 12, 56).

The spatial and temporal distribution of PM₂.₅ concentrations across Thailand during the haze season (January–April 2025) revealed pronounced regional and seasonal heterogeneity. The highest pollution burdens were consistently observed in the Northern region, followed by the Northeastern and Central regions, whereas the Southern region experienced comparatively lower concentrations. PM₂.₅ levels increased sharply from January to February and remained persistently elevated through March in the Northern and Northeastern regions, indicating sustained atmospheric accumulation during the peak haze period. This pattern likely reflects the combined influence of biomass burning, forest fires, transboundary pollution, and unfavorable meteorological conditions that limit pollutant dispersion (1, 3, 4). Within this context, environmental factors were associated with differences in symptom risk. Our findings showed elevated odds of respiratory and eye symptoms among suburban residents and increased odds of dermatological symptoms among urban residents, suggesting that differences in emission sources and exposure dynamics may critically influence the type and distribution of air pollution related health outcomes. Urban areas are typically dominated by emissions from traffic, industrial activities, and construction, resulting in relatively continuous, combustion-related air pollution throughout the year. In contrast, suburban environments are influenced by a combination of traffic emissions, small-scale industry, and intermittent open burning, producing a more heterogeneous and transitional pollution profile shaped by both urban and rural sources (14, 24). Such heterogeneity in exposure composition and intensity may contribute to distinct symptom patterns during air pollution episodes, particularly under prolonged haze conditions. Consistent with our findings, several previous studies have reported that PM₂.₅ exposure is strongly associated with increased mortality and adverse health outcomes, particularly respiratory effects, across different levels of urbanization (57–59). Evidence further suggests that acute health effects of PM₂.₅, especially respiratory outcomes, are often more pronounced or more consistently observed in urban and suburban populations than in rural areas. This pattern may reflect higher exposure to traffic- and industry-related co-pollutants, greater population density, and differences in population vulnerability. Supporting this interpretation, Liu et al. (2016) (60) demonstrated that short-term increases in PM₂.₅ and other air pollutants were significantly associated with increased emergency room visits for respiratory diseases in both urban and suburban settings, reinforcing emergency room utilization as a sensitive indicator of acute air pollution related health effects.

Importantly, our results further indicate that higher ambient PM₂.₅ concentrations were strongly associated with acute health symptoms, particularly respiratory and dermatological outcomes, with evidence of a dose–response relationship. These findings are consistent with a growing body of epidemiological evidence from Thailand demonstrating adverse health outcomes associated with ambient air pollution across different regions, populations, and exposure periods. Surit et al. (2023) (61) reported that deteriorating air quality was associated with increased emergency department visits, particularly for acute respiratory diseases, while Varapongpisan et al. (2022) (62) found that higher ambient air pollutant concentrations were associated with increased outpatient visits for respiratory diseases during seasonal haze episodes in Chiang Mai. More recently, Varopichetsan et al. (2025) (6) reported an association between short-term PM₂.₅ exposure and asthma exacerbations in a large population-based study in southern Thailand. Jarernwong et al. (2023) (63) further demonstrated spatial correlations between PM₂.₅ concentrations and multiple health outcomes, including conjunctivitis, dermatitis, stroke, and lung cancer, supporting the multisystem symptom profile observed in the present study. Consistent with our high prevalence of ocular symptoms, Kausar et al. (2024) (13) reported greater odds of ocular redness, watering, and dryness during periods of elevated PM₂.₅ exposure among adults in Chiang Mai. Additionally, evidence from longer-term and national studies further supports the broader population health relevance of these findings. Sapbamrer et al. (2) reported associations between long-term ambient air pollution and COPD and lung cancer incidence in Upper Northern Thailand, while Punnasiri et al. (2025) (5), using data from 31 provinces, demonstrated associations between short-term exposure to multiple ambient air pollutants and mortality in developing Thailand’s national Air Quality Health Index. Together, these studies indicate that the health burden associated with ambient air pollution in Thailand encompasses acute symptoms, healthcare utilization, disease exacerbation, chronic respiratory outcomes, and mortality. The present nationwide study extends this evidence by demonstrating associations with multisystem acute symptoms across a geographically diverse population during the haze season, while simultaneously considering protective behavioral phenotypes and sociodemographic characteristics.

This study is among the largest nationwide investigations conducted in Thailand to simultaneously examine ambient PM₂.₅ exposure, protective behavioral phenotypes, and multisystem acute health symptoms during the haze season. Its major strengths include the large, geographically diverse sample representing most provinces across all major regions, the integration of individual-level survey data with province-specific ambient PM₂.₅ concentrations, and the application of mixed-effects models to account for provincial clustering. Furthermore, the use of latent class analysis enabled the identification of distinct protective behavioral phenotypes, providing a more realistic representation of real-world behavioral patterns and their associations with acute health outcomes than analyses focusing on individual protective practices alone.

Nevertheless, several limitations should be considered when interpreting the findings. First, the cross-sectional design precludes causal inference and does not permit determination of the temporal sequence between protective behaviors and symptom occurrence. Because protective behaviors and acute health symptoms were assessed during the same recall period, reverse causation cannot be excluded. Individuals who experienced respiratory, eye, dermatological, or neurological symptoms may have subsequently adopted more protective behaviors, such as wearing masks, reducing outdoor activities, or improving indoor air quality. Consequently, the identified behavioral phenotypes may partially reflect prior symptom experience rather than functioning solely as determinants of symptom occurrence. Second, acute health symptoms and protective behaviors were self-reported and may therefore be subject to recall or reporting bias, although the use of standardized questionnaires and trained interviewers likely reduced random misclassification. Third, ambient PM₂.₅ exposure was assessed using province-level monthly average concentrations, which provided a standardized exposure metric for this nationwide study but could not capture individual variability arising from commuting patterns, occupational activities, housing characteristics, indoor environments, ventilation, or daily time–activity patterns. Consequently, some degree of non-differential exposure misclassification is likely, which would generally bias effect estimate toward the null. Therefore, the observed associations may underestimate the true relationship between ambient PM₂.₅ exposure and acute health symptoms. Finally, although the multivariable models adjusted for important sociodemographic and behavioral factors, residual confounding cannot be excluded because meteorological variables (e.g., temperature, relative humidity, rainfall, wind speed, atmospheric pressure, and atmospheric stability) and gaseous co-pollutants (e.g., NO₂, SO₂, O₃, and CO) were not incorporated into the analyses owing to the lack of consistently available nationwide spatiotemporally harmonized data. These environmental factors may independently influence both ambient PM₂.₅ concentrations and acute health symptoms, and their omission may have resulted in residual confounding.

Future research should employ longitudinal or prospective study designs to establish temporal relationships and strengthen causal inference regarding haze exposure, protective behavioral adaptation, and health outcomes. Incorporating higher-resolution exposure models, residential geocoding, personal exposure monitoring, indoor air quality measurements, meteorological variables, and co-pollutants would improve exposure characterization and enable more precise estimation of the independent health effects of PM₂.₅. In addition, investigations of PM₂.₅ chemical composition and emission sources, together with qualitative and implementation research exploring structural, cultural, and economic barriers to protective behavior adoption, would provide valuable evidence for developing more equitable, effective, and sustainable haze mitigation and public health strategies.

## 5 Conclusion

This nationwide cross-sectional study found that ambient PM₂.₅ exposure, educational, behavioral, and social factors were associated with acute health symptoms during Thailand’s haze season. Although ambient PM₂.₅ concentrations exceeded international guideline values across much of the country, participants exhibited substantial heterogeneity in protective behavioral patterns, which was associated with differences in symptom occurrence. Respiratory symptoms were the most frequently reported, followed by eye, dermatological, and neurological symptoms, highlighting the multisystem health burden observed during the haze season. The identification of three distinct protective behavioral phenotypes (compliers, non-compliers, and neutrals) further demonstrated that participants with different behavioral patterns experienced different odds of acute health symptoms despite comparable ambient PM₂.₅ exposure. Compared with compliers, individuals classified as non-compliers or neutrals had significantly greater odds of respiratory and neurological symptoms. These findings suggest that protective behavioral patterns may be associated with differences in vulnerability to haze-related health symptoms, although the cross-sectional design does not permit conclusions regarding causality.

Acute health symptoms were also associated with gender, age, educational attainment, comorbidity status, lifestyle behaviors, residential setting, and community health engagement. Women, younger individuals, those with underlying health conditions had greater odds of reporting symptoms, whereas village health volunteers had lower odds of symptoms than non-volunteers. Differences in symptom patterns across urban, suburban, and rural areas further suggest that environmental and contextual factors may contribute to variations in haze-related health outcomes.

Collectively, these findings indicate that interventions focused solely on increasing individual awareness are unlikely to be sufficient. Effective mitigation of haze-related health impacts requires integrated approaches that combine emission reduction, geographically tailored air quality management, behaviorally informed risk communication, and strengthened community health systems. Structural interventions, such as stricter regulation of biomass burning, improved urban and transport planning, and expanded access to affordable protective technologies, are essential complements to individual-level behavioral change. Leveraging trusted community actors, particularly village health volunteers, represents a critical opportunity to translate risk information into sustained protective action, given their established roles in health promotion, surveillance, and community engagement. Addressing economic, infrastructural, and contextual barriers that limit adoption of effective protective behaviors, especially among younger adults and non-complier groups, will be central to enhancing population resilience during recurrent haze episodes. Importantly, incorporating behavioral phenotypes into public health planning enables policymakers to move beyond one-size-fits-all messaging toward targeted, equity-oriented strategies that align interventions with population heterogeneity in risk perception, health engagement, and adaptive capacity. Such an approach has the potential to substantially reduce the acute health burden of air pollution in Thailand and to inform scalable haze-mitigation frameworks applicable to other settings facing recurrent particulate pollution.

## Data Availability

The raw data supporting the conclusions of this article will be made available by the authors, without undue reservation.
