# Supplementary material for: Health inequalities in PM₂.₅-related symptoms during Thailand’s haze season: the role of education, sociodemographic, lifestyle, protective behaviors, and environmental factors
Source: Front Public Health. 2026 Oct 1;14:1899785. doi: 10.3389/fpubh.2026.1899785 (PMC13668745; doi:10.3389/fpubh.2026.1899785)
Supplement: Supplementary file 1 [file Data_Sheet_1.docx]

**Supplementary material**

**Supplementary table S1.** The distribution of participants by province, Thailand

| **Province** | **Participants** |
| --- | --- |
| Chiang Mai | 205 |
| Mae Hong Son | 1 |
| Chiang Rai | 73 |
| Lampang | 8 |
| Phayao | 51 |
| Nan | 103 |
| Tak | 4 |
| Sukhothai | 15 |
| Phitsanulok | 132 |
| Kamphaeng Phet | 2 |
| Phetchabun | 54 |
| Phichit | 14 |
| Kalasin | 65 |
| Khon Kaen | 125 |
| Chaiyaphum | 87 |
| Nakhon Phanom | 65 |
| Nakhon Ratchasima | 151 |
| Buri Ram | 61 |
| Maha Sarakham | 2 |
| Mukdahan | 81 |
| Yasothon | 118 |
| Roi Et | 77 |
| Loei | 1 |
| Si Saket | 21 |
| Sakon Nakhon | 105 |
| Surin | 110 |
| Nong Khai | 42 |
| Nong Bua Lamphu | 43 |
| Udon Thani | 119 |
| Ubon Ratchathani | 49 |
| Amnat Charoen | 2 |
| Bangkok | 114 |
| Uthai Thani | 34 |
| Chainat | 79 |
| Nakhon Sawan | 17 |
| Nonthaburi | 91 |
| Pathum Thani | 45 |
| Phra Nakhon Si Ayutthaya | 115 |
| Lopburi | 27 |
| Samut Prakan | 81 |
| Samut Songkhram | 1 |
| Samut Sakhon | 93 |
| Saraburi | 47 |
| Singburi | 27 |
| Ang Thong | 32 |
| Chonburi | 37 |
| Rayong | 92 |
| Chanthaburi | 8 |
| Trat | 82 |
| Nakhon Nayok | 24 |
| Chachoengsao | 7 |
| Prachinburi | 42 |
| Sa Kaeo | 29 |
| Kanchanaburi | 68 |
| Prachuap Khiri Khan | 63 |
| Suphanburi | 98 |
| Phetchaburi | 27 |
| Nakhon Pathom | 8 |
| Ratchaburi | 50 |
| Chumphon | 4 |
| Trang | 30 |
| Nakhon Si Thammarat | 10 |
| Narathiwat | 116 |
| Pattani | 33 |
| Phang Nga | 1 |
| Phatthalung | 16 |
| Phuket | 24 |
| Yala | 36 |
| Songkhla | 81 |
| Satun | 94 |
| Surat Thani | 47 |

**Supplementary table S2.** The latent class analysis model fit statistics

| **Number of classes** | **Sample size** | **Log-likelihood** | **p-value, log-likelihood ratio tests** | **Akaike's information criteria** | **Bayesian information criteria** | **Entropy** | **Degree of freedom** |
| --- | --- | --- | --- | --- | --- | --- | --- |
| 1 | 3,916 | -71,056 | (reference) | 142,188.77 | 142,427.13 | NA | NA |
| 2 | 3,916 | -63,315 | 0.333 | 126,783.20 | 127,266.21 | 0.961 | 39 |
| 3 | 3,916 | -58,725 | <0.001 | 117,681.94 | 118,409.59 | 0.900 | 39 |
| 4 | 3,916 | -56,978 | <0.001 | 114,265.72 | 115,238.00 | 0.901 | 39 |
| 5 | 3,916 | -56,138 | <0.001 | 112,664.42 | 113,881.35 | 0.905 | 39 |

**Supplementary table S3.** The latent class analysis model class probability statistics across different numbers of classes

| **LCA model** | **Class probability (95% CIs)** |
| --- | --- |
| **Two-class model** |  |
| Class 1 | 0.79 (0.78, 0.81) |
| Class 2 | 0.21 (0.19, 0.22) |
| **Three-class model** |  |
| Class 1 | 0.34 (0.32, 0.36) |
| Class 2 | 0.48 (0.46, 0.50) |
| Class 3 | 0.18 (0.17, 0.20) |
| **Four-class model** |  |
| Class 1 | 0.16 (0.15, 0.18) |
| Class 2 | 0.47 (0.45, 0.49) |
| Class 3 | 0.18 (0.16, 0.20) |
| Class 4 | 0.19 (0.18, 0.20) |
| **Five-class model** |  |
| Class 1 | 0.07 (0.06, 0.08) |
| Class 2 | 0.31 (0.29, 0.34) |
| Class 3 | 0.28 (0.26, 0.30) |
| Class 4 | 0.16 (0.15, 0.18) |
| Class 5 | 0.17 (0.16, 0.19) |

**Supplementary table S4.** Variance inflation factor (VIF) for each determinant used in the mixed-effect logistic regression models

| **Determinants** | | **VIF** |
| --- | --- | --- |
| **Sociodemographic determinants** | |  |
|  | Gender | 1.30 |
|  | Age | 1.91 |
|  | Monthly income bracket | 1.53 |
|  | Education level | 2.14 |
|  | Co-morbidity | 1.25 |
| **Lifestyle and protective behavior determinants** | |  |
|  | Smoking | 1.33 |
|  | Alcohol consumption | 1.23 |
|  | Village health volunteer | 1.29 |
|  | Routine health checkup | 1.18 |
|  | Mask type use | 1.04 |
|  | Behavior classification | 1.07 |
| **Environmental determinants** | |  |
|  | Residential area | 1.13 |
|  | Monthly PM_2.5_ levels | 1.03 |
| **Mean VIF** | | 1.34 |

**Supplementary table S5.** Health behavior of participants during haze season in Thailand

| **Items** | **n (%)** | | |
| --- | --- | --- | --- |
|  | **Never** | **Sometimes** | **Always** |
| Clean indoor areas frequently | 110 (2.8) | 1,295 (33.1) | 2,511 (64.1)^a^ |
| Avoid smoking outdoors | 628 (16.0) | 778 (19.9) | 2,510 (64.1)^a^ |
| Avoid smoking indoors | 640 (16.3) | 771 (19.7) | 2,505 (64.0)^a^ |
| Avoid open waste burning | 436 (11.1) | 1,300 (33.2) | 2,180 (55.7) |
| Turn off the car engine when parked | 392 (10.0) | 1,541 (39.4) | 1,983 (50.6) |
| Keep windows closed | 307 (7.8) | 1,680 (42.9) | 1,929 (49.3) |
| Wear a mask outdoors | 115 (2.9) | 1,921 (49.1) | 1,880 (48.0) |
| Use an electric stove instead of coal | 466 (11.9) | 1,605 (41.0) | 1,845 (47.1) |
| Buy certified pollution-free products | 600 (15.3) | 1,836 (46.9) | 1,480 (37.8) |
| Avoid outdoor activities | 322 (8.2) | 2,173 (55.5) | 1,421 (36.3) |
| Postpone travel to polluted areas | 699 (17.8) | 2,165 (55.3) | 1,052 (26.9) |
| Avoid grilling food | 683 (17.4) | 2,313 (59.1) | 920 (23.5) |
| Work from home during haze season | 1,433 (36.6) | 1,591 (40.6) | 892 (22.8) |
| Use a home air purifier | 1,887 (48.2) | 1,160 (29.6) | 869 (22.2) |
| Use a bicycle for transportation | 1,201 (30.7) | 1,971 (50.3) | 744 (19.0) |
| Use a car air purifier | 2,013 (51.4) | 1,179 (30.1) | 724 (18.5) |
| Use public transportation | 1,400 (35.8) | 1,827 (46.7) | 689 (17.6)^b^ |
| Wear a mask indoors | 1,539 (39.3) | 1,686 (43.1) | 691 (17.6)^b^ |
| Use an electric vehicle | 2,265 (57.8) | 1,108 (28.3) | 543 (13.9)^b^ |

^a^ Top 3 questions participants answered “always”; ^b^ Bottom 3 questions participants answered “always”

**Supplementary table S6.** The sensitivity analysis of each mixed-effect logistic regression models with PM_2.5_ specification as linear and restricted cubic splines terms demonstrated by log-likelihood terms and tests of log-likelihood ratios

| **Outcome** | **PM 2.5 transformation** | | **p-value** |
| --- | --- | --- | --- |
|  | Linear | 4-degree of freedom restricted cubic spline model |  |
| Respiratory | -1658.80 | -1654.58 | 0.015 |
| Eye | -2409.33 | -2407.42 | 0.149 |
| Dermatological | -2327.03 | -2326.68 | 0.704 |
| Neurological | -2322.51 | -2321.48 | 0.357 |

**Supplementary figure S1.** Anscombe residual for mixed-effect logistic regression models

**Supplementary figure S2.** Random intercepts distribution for mixed-effect logistic regression models, visualized by histograms

**Supplementary figure S3.** Random intercepts distribution for mixed-effect logistic regression models, visualized by quantile-quantile plots
